# Synthesis, crystallographic analysis and Hirshfeld surface analysis of 4-bromo-2-{[2-(5-bromo-2-nitro­phen­yl)hydrazin-1-yl­idene]meth­yl}-5-fluoro­phenol

**DOI:** 10.1107/S2056989018014627

**Published:** 2018-10-19

**Authors:** Mavise Yaman, Ercan Aydemir, Necmi Dege, Erbil Agar, Turganbay S. Iskenderov

**Affiliations:** a Ondokuz Mayıs University, Faculty of Arts and Sciences, Department of Physics, 55139, Kurupelit, Samsun, Turkey; b Ondokuz Mayıs University, Faculty of Arts and Sciences, Department of Chemistry, 55139, Samsun, Turkey; c Ministry of Forestry and Water Affairs , 11th Regional Directorate, 55030, Ilkadım-Samsun, Turkey; d Taras Shevchenko National University of Kyiv, Department of Chemistry, 64, Vladimirska Str., Kiev 01601, Ukraine

**Keywords:** crystal structure, Hirshfeld surface, Hydrazone, crystal structure, hydrogen bonding, 5-bromo-4-fluoro-2-hy­droxy­benzaldehyde

## Abstract

The title compound, C_13_H_8_Br_2_FN_3_O_3_, is nearly planar with a dihedral angle of 10.6 (4)° between the two benzene rings. Intra­molecular N—H⋯O and O—H⋯N hydrogen bonds occur. In the crystal, the mol­ecules are linked by weak C—H⋯O and C—H⋯Br hydrogen bonds. The roles of the inter­molecular inter­actions in the crystal packing were clarified using Hirshfeld surface analysis.

## Abstract 2

The title compound is nearly planar with a dihedral angle of 10.6 (4)° between the two benzene rings.

## Chemical context   

Hydrazones, the most important derivatives of carboxaldehyde, are widely used both in organic synthesis and in industrial work because of their reaction abilities, such as ring closing, oxidation-reduction, replacement reactions and coupling (Öztürk *et al.*, 2003 ▸). They are generally considered to be useful starting materials for the production of pharmaceuticals, pesticides, textile dyestuffs as well as compounds that serve as stabilizers and inhibitors in photography (Kaban & Ocal, 1993 ▸). In addition, they exhibit a wide range of applications in the fields of biology, optics, catalysis and analytical chemistry. Their broad spectrum of biological activities includes anti­microbial, anti­fungal, anti­viral, anti­tumor, anti-HIV, anti-inflammatory, anti­neoplastic and analgesic activities (Sudheer *et al.*, 2015 ▸; Soujanya & Rajitha, 2017 ▸). Hydrazone-based mol­ecular switches, metalloassemblies and sensors have also been developed (Sudheer *et al.*, 2015 ▸). Unlike oximes (Sliva *et al.*, 1997 ▸; Penkova *et al.*, 2010 ▸; Pavlishchuk *et al.*, 2010 ▸), hydrazones are mostly obtained as a mixture of *E* and *Z* isomers and both isomers are generally weak acids (Mori *et al.*, 2015 ▸). Tautomerism between the isomers might also occur in the case of the hydrazone and azo forms (Aydemir & Kaban, 2018 ▸). In this study, the structure of the newly synthesized compound has been evaluated by spectroscopic techniques. In view of this, in order to obtain information about the stereochemistry of the mol­ecule and to confirm the assigned structure, X-ray analysis of the title compound was undertaken.
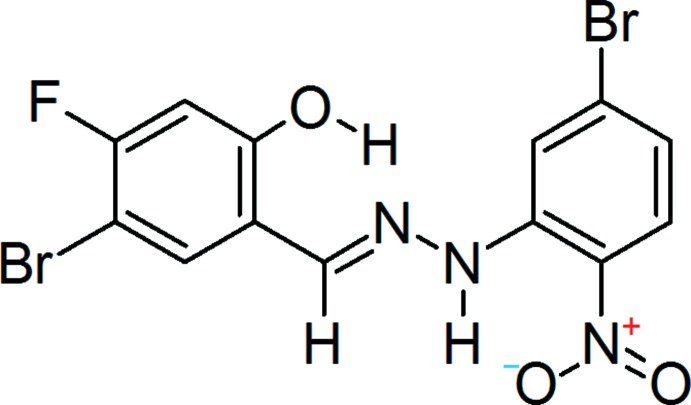


## Structural commentary   

**Figure 1:**
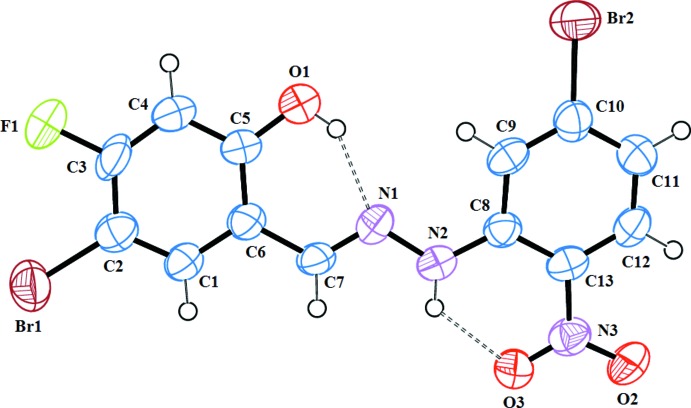
An *ORTEP* view of 4-bromo-2-{[2-(5-bromo-2-nitro­phen­yl)hydrazin-1-yl­idene]meth­yl}-5-fluoro­phenol. Displacement ellipsoids are drawn at the 50% probability level.

The mol­ecular structure of the title compound is illustrated in Fig. 1 ▸. The dihedral angle between the aromatic rings is 10.6 (4)°. The N1—N2 and N2–C8 bond lengths are 1.368 (7) and 1.374 (8) Å, respectively. The C13–N3 bond [1.451 (8) Å] in the nitro group is close to the standard value for this type of bond (Allen *et al.*, 1987 ▸). Intra­molecular N2—H2⋯O3 and O1—H1⋯N1 hydrogen-bonding inter­actions (Table 1 ▸) occur.

## Supra­molecular features   

**Figure 2:**
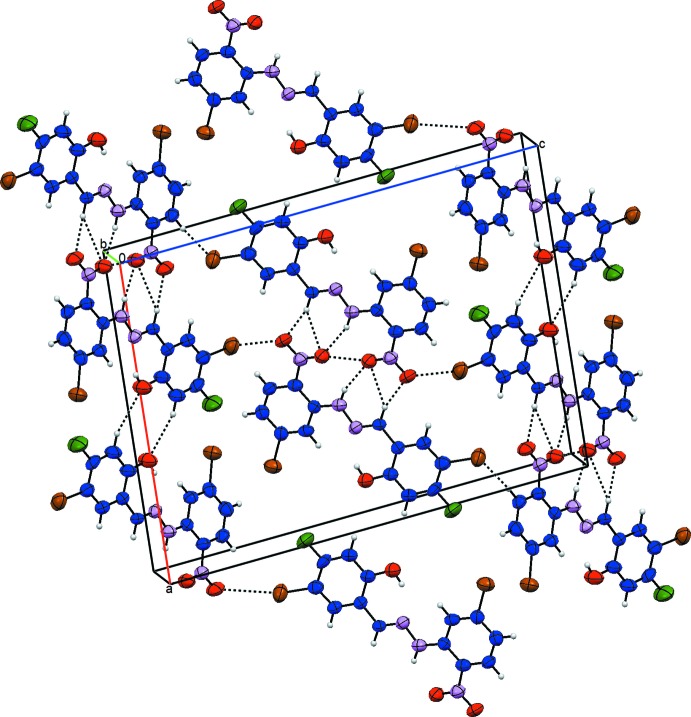
The view of the crystal packing of the title compound.

In the crystal, the mol­ecules are linked by weak C—H⋯O and C—H⋯Br hydrogen bonds (Table 1 ▸, Fig. 2 ▸).

## Hirshfeld surface analysis   

**Figure 3:**
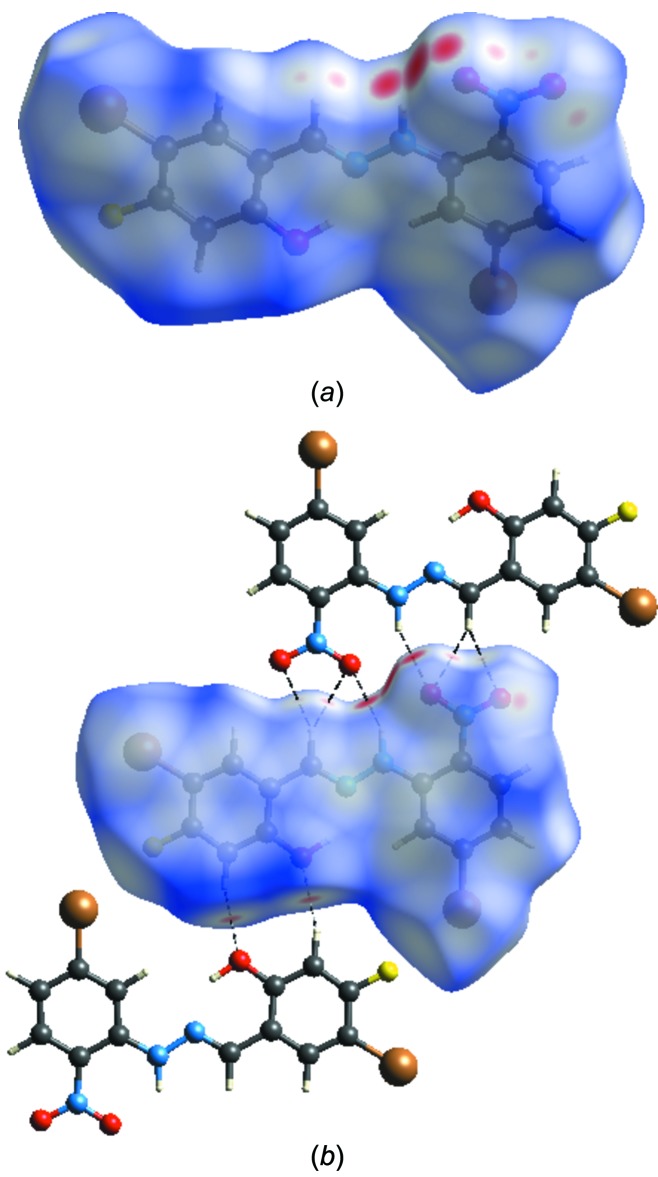
Views of the Hirshfeld surface of the title compound mapped over *d*_norm_.

A Hirshfeld surface analysis was performed to qu­antify the nature of the inter­molecular inter­actions. The Hirshfeld surfaces were generated using *CrystalExplorer17.5* (Turner *et al.*, 2017 ▸) using a standard (high) surface resolution. Fig. 3 ▸ shows the Hirshfeld surfaces mapped over *d*_norm_ in the range −0.2247 (red) to 1.3787 (blue) a.u. If the value of *d*_norm_ is negative, the inter­molecular contacts are shorter than the van der Waals radius; these are shown as red regions. A positive value of *d*_norm_, shown in blue, indicates that the inter­molecular contacts are longer than the van der Waals radius (Şen *et al.*, 2017 ▸). The red regions on the *d*_norm_ surface correspond to C—H⋯O hydrogen-bonding inter­actions, which comprise 20.2% of the total Hirshfeld surfaces.

**Figure 4:**
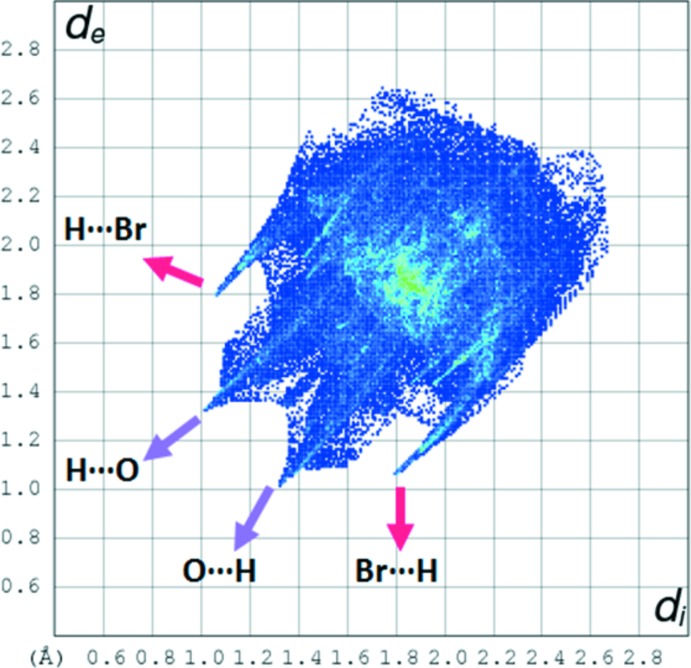
Fingerprint plot of the title compound showing all inter­actions.

**Figure 5:**
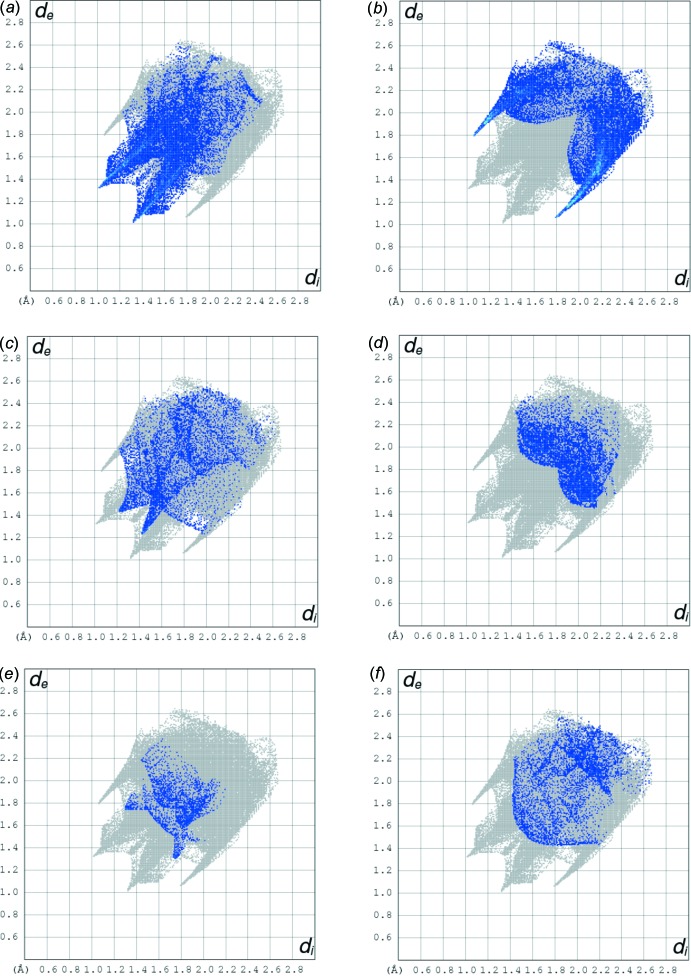
Two-dimensional fingerprint plots with a *d*_norm_ view of the (*a*) O⋯H/H⋯O (20.2%), (*b*) Br⋯H/H⋯Br (21.7%), (*c*) F⋯H/H⋯F (7.4%), (*d*) C⋯H/H⋯C (9.7%), (*e*) N⋯H/H⋯N (3.3%) and (*f*) H⋯H (6.0%) contacts in the title compound.

**Figure 6:**
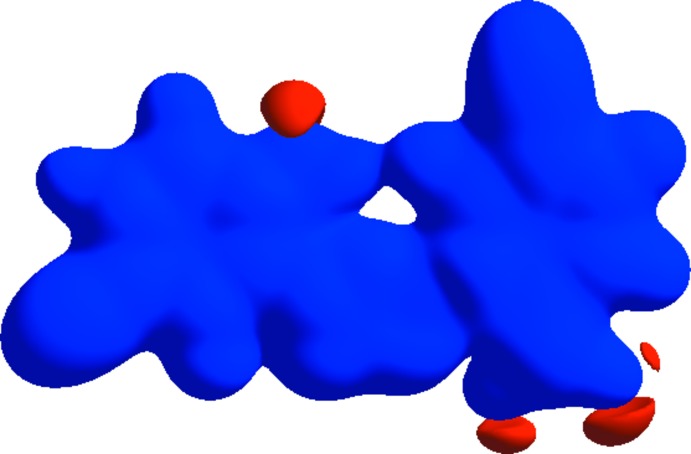
A view of the three-dimensional Hirshfeld surface of the title compound plotted over electrostatic potential.

The two-dimensional fingerprint (FP) plots are used to analyse significant differences between the inter­molecular inter­action patterns (Gumus *et al.*, 2018 ▸; Kansız & Dege, 2018 ▸; Kansiz *et al.*, 2018 ▸). Fig. 4 ▸ represents the FP plot for the sum of the contacts contributing to the Hirshfeld surface displayed in normal mode. In Fig. 5 ▸ distinct spikes indicate different inter­actions between two adjacent mol­ecules in the crystal structure. The contribution from the Br⋯H/H⋯Br contacts make the largest (21.7%) to the Hirshfeld surface (Fig. 5 ▸ *b*). The 20.2% contribution from the O—H⋯O hydrogen bond is seen as a pair of sharp spikes at *d*_e_ + *d*_i_ = 2.3 Å) in Fig. 5 ▸ *a*. The distribution of positive and negative potential over the Hirshfeld surface is represented in Fig. 6 ▸ (positive electrostatic potential shown in blue region and negative electrostatic potential in red).

## Database survey   

There are no direct precedents for the structure of C_13_H_8_Br_2_FN_3_O_3_ in the crystallographic literature (CSD, version 5.39, update of May 2018; Groom *et al.*, 2016 ▸) but some similar structures including 2-nitro­phenyl­hydrazine have been reported. All geometric parameters in the title compound agree well with those reported in the literature with the N1—N2 and N2—C8 bond distances being comparable to those in *N*-(4-chloro-2-nitro­phen­yl)-*N*′-methyl-*N*-(quinolin-4-yl­meth­yl­ene)hydrazine [1.367 (2) and 1.386 (3) Å; Karadayı *et al.*, 2005 ▸] and *N*-(4-bromo-2-nitro­phen­yl)-*N*-methyl-*N*′-(quinolin-4-yl­methyl­ene)hydrazine [1.359 (3) and 1.393 (4) Å; Öztürk *et al.*, 2003 ▸].

## Synthesis and crystallization   

**Figure 7:**
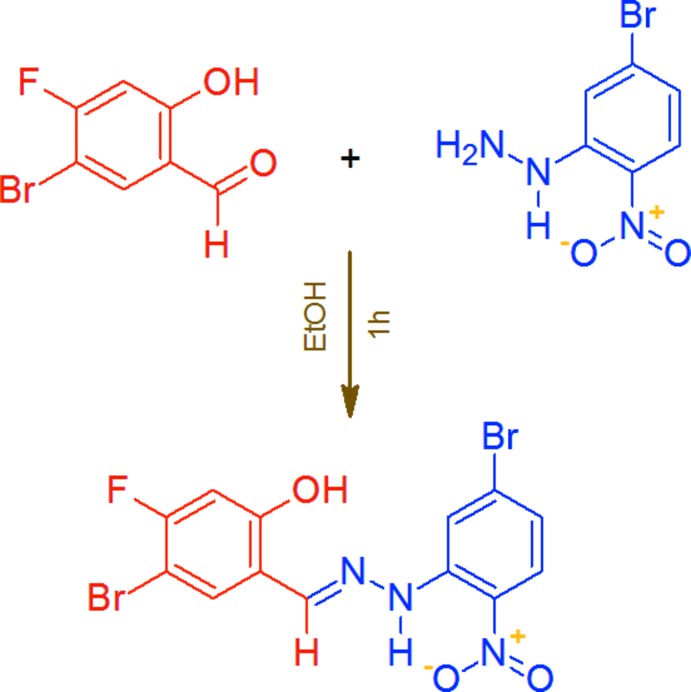
The synthesis of the title compound.

5-Bromo-4-fluoro-2-hy­droxy­benzaldehyde (0.5 mmol) was dissolved in hot absolute ethanol (10 mL) and an equimolar amount of 5-bromo-2-nitro­phenyl­hydrazine, dissolved in a minimum volume of absolute ethanol, was slowly added. The product appeared in the first minute. The reaction mixture was refluxed for an additional hour to complete the condensation and then allowed to cool in room temperature. The separated solid was then filtered and washed with ethanol and diethyl ether. The crude product was recrystallized from toluene as pink needle-shaped crystals, 96% yield, m.p. 569–570 K (dec.). The reaction scheme is shown in Fig. 7 ▸. UV (CHCl_3_): λ_max_ 340, 430 nm; IR (KBr): υ 3610 (–OH), 3285 and 1155 (N—H), 3120–2985 (=C—H), 2915 (C—H), 1608 (C=N), 1558 (C=C), 1515 (N—N), 1475 and 1310 (N=O), 1195, 690 and 665 (C—*X*) cm^−1^; MS (ESI^+^): 434.01 ([*M* + H]^+^, C_13_H_8_Br_2_FN_3_O_3_; calculated 433.03).

## Refinement   

Crystal data, data collection and structure refinement details are summarized in Table 2 ▸. The C-bound hydrogen atoms were positioned geometrically and refined using a riding model: C—H = 0.93–0.97 Å with *U*_iso_(H) = 1.2*U*_eq_(C)

## Supplementary Material

Crystal structure: contains datablock(s) I. DOI: 10.1107/S2056989018014627/xu5944sup1.cif

Structure factors: contains datablock(s) I. DOI: 10.1107/S2056989018014627/xu5944Isup2.hkl

Supporting information file. DOI: 10.1107/S2056989018014627/xu5944Isup3.cml

CCDC reference: 1864935

Additional supporting information:  crystallographic information; 3D view; checkCIF report

## Figures and Tables

**Table 1:** Hydrogen-bond geometry (Å, °)

*D*—H⋯*A*	*D*—H	H⋯*A*	*D*⋯*A*	*D*—H⋯*A*
O1—H1⋯N1	0.82	1.91	2.631 (7)	146
N2—H2⋯O3	0.86	2.01	2.619 (7)	127
N2—H2⋯O3^i^	0.86	2.50	3.293 (7)	155
C4—H4⋯O1^ii^	0.93	2.60	3.494 (8)	162
C7—H7⋯O3^i^	0.93	2.66	3.461 (7)	145
C12—H12⋯Br1^iii^	0.93	3.02	3.908 (7)	161

Symmetry codes: (i) 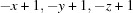; (ii) 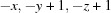; (iii) 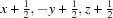.

**Table 2:** Experimental details

Crystal data	
Chemical formula	C_13_H_8_Br_2_FN_3_O_3_
*M*_r_	433.04
Crystal system, space group	Monoclinic, *P*2_1_/*n*
Temperature (K)	296
*a*, *b*, *c* (Å)	16.1360 (14), 4.1745 (3), 21.468 (2)
β (°)	95.026 (7)
*V* (Å^3^)	1440.5 (2)
*Z*	4
Radiation type	Mo *K*α
μ (mm^−1^)	5.65
Crystal size (mm)	0.46 × 0.17 × 0.02

Data collection	
Diffractometer	Stoe IPDS 2
Absorption correction	Integration (*X-RED32*; Stoe & Cie, 2002 ▸)
*T*_min_, *T*_max_	0.296, 0.883
No. of measured, independent and observed [*I* > 2σ(*I*)] reflections	9546, 2775, 1270
*R*_int_	0.113
(sin θ/λ)_max_ (Å^−1^)	0.617

Refinement	
*R*[*F* ^2^ > 2σ(*F* ^2^)], *wR*(*F* ^2^), *S*	0.051, 0.098, 0.84
No. of reflections	2775
No. of parameters	200
H-atom treatment	H-atom parameters constrained
Δρ_max_, Δρ_min_ (e Å^−3^)	0.42, −0.28

Computer programs: *X-AREA* and *X-RED* (Stoe & Cie, 2002 ▸), *SHELXT2014* (Sheldrick, 2015*a* ▸), *SHELXL2017* (Sheldrick, 2015*b* ▸), *ORTEP-3 for Windows* and *WinGX* (Farrugia, 2012 ▸) and *PLATON* (Spek, 2009 ▸).

## supplementary crystallographic information

### Crystal data

**Table:**

C_13_H_8_Br_2_FN_3_O_3_	*F*(000) = 840
*M**_r_* = 433.04	*D*_x_ = 1.997 Mg m^−^^3^
Monoclinic, *P*2_1_/*n*	Mo *K*α radiation, λ = 0.71073 Å
*a* = 16.1360(14) Å	Cell parameters from 5914 reflections
*b* = 4.1745 (3) Å	θ = 1.5–29.7°
*c* = 21.468 (2) Å	µ = 5.65 mm^−^^1^
β = 95.026 (7)°	*T* = 296 K
*V* = 1440.5 (2) Å^3^	Needle, pink
*Z* = 4	0.46 × 0.17 × 0.02 mm

### Data collection

**Table:**

Stoe IPDS 2 diffractometer	2775 independent reflections
Radiation source: sealed X-ray tube, 12 x 0.4 mm long-fine focus	1270 reflections with *I* > 2σ(*I*)
Detector resolution: 6.67 pixels mm^-1^	*R*_int_ = 0.113
rotation method scans	θ_max_ = 26.0°, θ_min_ = 1.5°
Absorption correction: integration (X-RED32; Stoe & Cie, 2002)	*h* = −19→19
*T*_min_ = 0.296, *T*_max_ = 0.883	*k* = −4→5
9546 measured reflections	*l* = −26→26

### Refinement

**Table:**

Refinement on *F*^2^	0 restraints
Least-squares matrix: full	Hydrogen site location: inferred from neighbouring sites
*R*[*F*^2^ > 2σ(*F*^2^)] = 0.051	H-atom parameters constrained
*wR*(*F*^2^) = 0.098	*w* = 1/[σ^2^(*F*_o_^2^) + (0.0267*P*)^2^] where *P* = (*F*_o_^2^ + 2*F*_c_^2^)/3
*S* = 0.84	(Δ/σ)_max_ < 0.001
2775 reflections	Δρ_max_ = 0.42 e Å^−^^3^
200 parameters	Δρ_min_ = −0.28 e Å^−^^3^

### Special details

**Table:**

Geometry. All esds (except the esd in the dihedral angle between two l.s. planes) are estimated using the full covariance matrix. The cell esds are taken into account individually in the estimation of esds in distances, angles and torsion angles; correlations between esds in cell parameters are only used when they are defined by crystal symmetry. An approximate (isotropic) treatment of cell esds is used for estimating esds involving l.s. planes.

### Fractional atomic coordinates and isotropic or equivalent isotropic displacement parameters (Å^2^)

**Table:**

	*x*	*y*	*z*	*U*_iso_*/*U*_eq_	
Br2	0.19136 (4)	−0.4176 (2)	0.67484 (4)	0.0714 (3)	
Br1	0.12607 (5)	1.1323 (3)	0.25304 (4)	0.0787 (3)	
O3	0.5159 (3)	0.2810 (14)	0.5457 (2)	0.0689 (16)	
F1	−0.0194 (2)	0.9500 (15)	0.3236 (2)	0.107 (2)	
N1	0.2772 (3)	0.3510 (15)	0.4968 (3)	0.0503 (14)	
N3	0.5145 (3)	0.0903 (18)	0.5895 (3)	0.0566 (15)	
N2	0.3538 (3)	0.2591 (14)	0.5232 (3)	0.0564 (18)	
H2	0.398154	0.325205	0.507843	0.068*	
O2	0.5792 (3)	−0.0104 (14)	0.6187 (2)	0.0800 (19)	
O1	0.1152 (3)	0.4263 (18)	0.4954 (3)	0.0892 (19)	
H1	0.161084	0.357911	0.508414	0.134*	
C8	0.3593 (4)	0.0605 (18)	0.5745 (3)	0.0470 (18)	
C1	0.1976 (4)	0.8061 (17)	0.3618 (3)	0.0496 (19)	
H1A	0.248599	0.848552	0.346371	0.060*	
C13	0.4351 (4)	−0.0228 (17)	0.6077 (3)	0.050 (2)	
C7	0.2755 (4)	0.5174 (18)	0.4473 (3)	0.050 (2)	
H7	0.324917	0.564799	0.430059	0.060*	
C11	0.3659 (4)	−0.3285 (19)	0.6826 (3)	0.062 (2)	
H11	0.367763	−0.454009	0.718527	0.074*	
C12	0.4369 (4)	−0.2110 (18)	0.6608 (3)	0.053 (2)	
H12	0.487920	−0.259040	0.682367	0.064*	
C6	0.1965 (4)	0.636 (2)	0.4169 (3)	0.0542 (19)	
C9	0.2876 (4)	−0.0681 (18)	0.5974 (3)	0.0497 (18)	
H9	0.236095	−0.024328	0.576206	0.060*	
C10	0.2909 (4)	−0.2535 (18)	0.6492 (3)	0.055 (2)	
C2	0.1272 (4)	0.913 (2)	0.3293 (3)	0.0569 (19)	
C5	0.1212 (4)	0.584 (2)	0.4414 (3)	0.067 (2)	
C4	0.0481 (4)	0.692 (2)	0.4105 (4)	0.078 (3)	
H4	−0.002785	0.660131	0.426706	0.093*	
C3	0.0533 (4)	0.849 (2)	0.3552 (3)	0.067 (2)	

### Atomic displacement parameters (Å^2^)

**Table:**

	*U*^11^	*U*^22^	*U*^33^	*U*^12^	*U*^13^	*U*^23^
Br2	0.0566 (5)	0.0778 (7)	0.0821 (6)	−0.0020 (5)	0.0197 (4)	0.0045 (5)
Br1	0.0781 (6)	0.0915 (8)	0.0632 (5)	−0.0096 (5)	−0.0115 (4)	0.0140 (5)
O3	0.053 (3)	0.084 (5)	0.070 (3)	0.002 (3)	0.007 (3)	0.023 (3)
F1	0.052 (2)	0.167 (6)	0.098 (3)	0.020 (3)	−0.010 (2)	0.037 (3)
N1	0.043 (3)	0.054 (5)	0.053 (4)	0.006 (3)	−0.002 (3)	−0.005 (3)
N3	0.046 (3)	0.066 (5)	0.058 (4)	−0.002 (4)	0.002 (3)	−0.006 (4)
N2	0.040 (3)	0.070 (5)	0.060 (4)	0.003 (3)	0.008 (3)	0.006 (3)
O2	0.040 (3)	0.116 (6)	0.082 (4)	0.010 (3)	−0.006 (3)	0.016 (3)
O1	0.052 (3)	0.136 (6)	0.080 (4)	0.010 (4)	0.011 (3)	0.042 (4)
C8	0.043 (4)	0.056 (5)	0.043 (4)	0.003 (4)	0.005 (3)	−0.004 (4)
C1	0.042 (4)	0.049 (6)	0.058 (5)	−0.001 (3)	0.002 (3)	0.000 (4)
C13	0.040 (4)	0.053 (6)	0.060 (5)	−0.004 (3)	0.007 (3)	−0.006 (4)
C7	0.033 (4)	0.068 (7)	0.048 (4)	−0.004 (3)	0.005 (3)	0.000 (4)
C11	0.064 (5)	0.069 (7)	0.053 (4)	0.007 (4)	0.010 (4)	0.009 (4)
C12	0.043 (4)	0.061 (6)	0.054 (4)	0.009 (4)	−0.004 (3)	−0.003 (4)
C6	0.041 (4)	0.073 (6)	0.047 (4)	−0.002 (4)	0.001 (3)	0.001 (4)
C9	0.041 (4)	0.047 (5)	0.061 (4)	0.007 (4)	0.007 (3)	−0.011 (4)
C10	0.059 (4)	0.056 (6)	0.049 (4)	0.012 (4)	0.006 (4)	−0.001 (4)
C2	0.049 (4)	0.059 (5)	0.062 (4)	−0.003 (4)	0.003 (3)	−0.007 (4)
C5	0.041 (4)	0.097 (7)	0.064 (5)	0.003 (5)	0.010 (4)	0.018 (5)
C4	0.042 (4)	0.124 (9)	0.069 (5)	0.008 (5)	0.012 (4)	0.015 (5)
C3	0.041 (4)	0.093 (7)	0.062 (5)	0.009 (4)	−0.019 (4)	−0.002 (5)

### Geometric parameters (Å, º)

**Table:**

Br2—C10	1.872 (7)	C1—H1A	0.9300
Br1—C2	1.874 (7)	C13—C12	1.383 (9)
O3—N3	1.234 (7)	C7—C6	1.465 (9)
F1—C3	1.369 (7)	C7—H7	0.9300
N1—C7	1.268 (8)	C11—C12	1.366 (9)
N1—N2	1.368 (7)	C11—C10	1.388 (9)
N3—O2	1.243 (7)	C11—H11	0.9300
N3—C13	1.451 (8)	C12—H12	0.9300
N2—C8	1.374 (8)	C6—C5	1.383 (8)
N2—H2	0.8600	C9—C10	1.353 (10)
O1—C5	1.343 (8)	C9—H9	0.9300
O1—H1	0.8200	C2—C3	1.386 (9)
C8—C9	1.402 (8)	C5—C4	1.378 (10)
C8—C13	1.406 (9)	C4—C3	1.363 (10)
C1—C2	1.356 (9)	C4—H4	0.9300
C1—C6	1.381 (9)		
			
C7—N1—N2	117.0 (5)	C11—C12—H12	119.0
O3—N3—O2	122.2 (6)	C13—C12—H12	119.0
O3—N3—C13	119.4 (6)	C1—C6—C5	119.0 (6)
O2—N3—C13	118.4 (6)	C1—C6—C7	118.6 (6)
N1—N2—C8	119.6 (5)	C5—C6—C7	122.4 (6)
N1—N2—H2	120.2	C10—C9—C8	122.3 (6)
C8—N2—H2	120.2	C10—C9—H9	118.9
C5—O1—H1	109.5	C8—C9—H9	118.9
N2—C8—C9	120.9 (6)	C9—C10—C11	121.6 (7)
N2—C8—C13	123.3 (6)	C9—C10—Br2	118.6 (5)
C9—C8—C13	115.8 (6)	C11—C10—Br2	119.8 (6)
C2—C1—C6	122.5 (6)	C1—C2—C3	116.2 (7)
C2—C1—H1A	118.8	C1—C2—Br1	123.6 (5)
C6—C1—H1A	118.8	C3—C2—Br1	120.1 (5)
C12—C13—C8	120.9 (6)	O1—C5—C4	116.9 (6)
C12—C13—N3	116.9 (6)	O1—C5—C6	122.5 (6)
C8—C13—N3	122.2 (6)	C4—C5—C6	120.5 (7)
N1—C7—C6	120.9 (6)	C3—C4—C5	117.5 (6)
N1—C7—H7	119.6	C3—C4—H4	121.2
C6—C7—H7	119.6	C5—C4—H4	121.2
C12—C11—C10	117.5 (7)	C4—C3—F1	117.7 (6)
C12—C11—H11	121.2	C4—C3—C2	124.2 (6)
C10—C11—H11	121.2	F1—C3—C2	118.1 (7)
C11—C12—C13	122.0 (6)		
			
C7—N1—N2—C8	−175.6 (6)	C13—C8—C9—C10	1.7 (10)
N1—N2—C8—C9	4.6 (10)	C8—C9—C10—C11	−0.3 (12)
N1—N2—C8—C13	−174.2 (6)	C8—C9—C10—Br2	−179.0 (5)
N2—C8—C13—C12	176.8 (7)	C12—C11—C10—C9	−0.7 (11)
C9—C8—C13—C12	−2.1 (10)	C12—C11—C10—Br2	178.0 (5)
N2—C8—C13—N3	−1.4 (10)	C6—C1—C2—C3	1.4 (12)
C9—C8—C13—N3	179.7 (6)	C6—C1—C2—Br1	−177.9 (6)
O3—N3—C13—C12	−173.8 (7)	C1—C6—C5—O1	−178.7 (8)
O2—N3—C13—C12	6.4 (9)	C7—C6—C5—O1	1.5 (13)
O3—N3—C13—C8	4.4 (10)	C1—C6—C5—C4	1.6 (13)
O2—N3—C13—C8	−175.3 (7)	C7—C6—C5—C4	−178.3 (8)
N2—N1—C7—C6	−177.9 (6)	O1—C5—C4—C3	−179.2 (9)
C10—C11—C12—C13	0.3 (11)	C6—C5—C4—C3	0.6 (14)
C8—C13—C12—C11	1.2 (11)	C5—C4—C3—F1	178.3 (8)
N3—C13—C12—C11	179.5 (7)	C5—C4—C3—C2	−1.9 (14)
C2—C1—C6—C5	−2.6 (12)	C1—C2—C3—C4	1.0 (13)
C2—C1—C6—C7	177.3 (7)	Br1—C2—C3—C4	−179.8 (8)
N1—C7—C6—C1	−177.6 (7)	C1—C2—C3—F1	−179.3 (7)
N1—C7—C6—C5	2.3 (12)	Br1—C2—C3—F1	−0.1 (11)
N2—C8—C9—C10	−177.2 (7)		

### Hydrogen-bond geometry (Å, º)

**Table:**

*D*—H···*A*	*D*—H	H···*A*	*D*···*A*	*D*—H···*A*
O1—H1···N1	0.82	1.91	2.631 (7)	146
N2—H2···O3	0.86	2.01	2.619 (7)	127
N2—H2···O3^i^	0.86	2.50	3.293 (7)	155
C4—H4···O1^ii^	0.93	2.60	3.494 (8)	162
C7—H7···O3^i^	0.93	2.66	3.461 (7)	145
C12—H12···Br1^iii^	0.93	3.02	3.908 (7)	161

Symmetry codes: (i) −*x*+1, −*y*+1, −*z*+1; (ii) −*x*, −*y*+1, −*z*+1; (iii) *x*+1/2, −*y*+1/2, *z*+1/2.
